# From basic research to the clinic: innovative therapies for ALS and FTD in the pipeline

**DOI:** 10.1186/s13024-020-00373-9

**Published:** 2020-06-01

**Authors:** Rajka Maria Liscic, Antonella Alberici, Nigel John Cairns, Maurizio Romano, Emanuele Buratti

**Affiliations:** 1 Department of Neurology, Johannes Kepler University, Linz, Austria; 2 School of Medicine, University of Osijek, Osijek, Croatia; 3 Neurology Unit, Department of Neurological Sciences and Vision, ASST-Spedali Civili-University of Brescia, Brescia, Italy; 4 College of Medicine and Health and Living Systems Institute, University of Exeter, Exeter, UK; 5 Department of Life Sciences, Via Valerio 28, University of Trieste, 34127 Trieste, Italy; 6 International Centre for Genetic Engineering and Biotechnology (ICGEB), Padriciano 99, 34149 Trieste, Italy

**Keywords:** ALS, Dementia FTLD, FTD, Genetics, Motor neuron disease TDP-43, Stem cell

## Abstract

Amyotrophic lateral sclerosis (ALS) and Frontotemporal Degeneration (FTD) are neurodegenerative disorders, related by deterioration of motor and cognitive functions and short survival. Aside from cases with an inherited pathogenic mutation, the causes of the disorders are still largely unknown and no effective treatment currently exists. It has been shown that FTD may coexist with ALS and this overlap occurs at clinical, genetic, and molecular levels. In this work, we review the main pathological aspects of these complex diseases and discuss how the integration of the novel pathogenic molecular insights and the analysis of molecular interaction networks among all the genetic players represents a critical step to shed light on discovering novel therapeutic strategies and possibly tailoring personalized medicine approaches to specific ALS and FTD patients.

## ALS and FTD: two sides of the same coin?

In principle, Amyotrophic lateral sclerosis (ALS) and Frontotemporal Degeneration (FTD) are two very different neurodegenerative diseases. ALS, whose incidence in Europe is 2.16 per 100000 person-years [1], is a progressive and ultimately fatal disease caused by progressive loss of motor neurons controlling voluntary muscles [2]. On the other hand, FTD, whose incidence in Europe is 3.5 per 100000 person-years [2], is a type of dementia characterized by atrophy of the frontal and temporal lobes, with composite clinical signs ranging from alterations in behavior and language to motor and cognitive dysfunctions [3]. More specifically, there is a general consensus that FTD consists of three distinct clinical syndromes: behavioral variant frontotemporal degeneration (bvFTD), non-fluent variant primary progressive aphasia (nfvPPA) and semantic variant primary progressive aphasia (svPPA) [4].

These pathologies were initially considered two independent clinical entities, because of heterogeneity at clinical and neuropathological levels [5–9]. Indeed, along this line, recent studies have outlined how these two diseases seem to be also characterized by different inflammatory profiles, with predominance of macrophage/microglia activation in ALS and astrocyte malfunctioning in FTD [10–12].

However, it is increasingly recognized that ALS is a multisystem disorder in which other non-motor (and in particular, cognitive and behavioral) impairments can be observed [13, 14], whereas, on the other side, FTD can be associated to signs of motor neuron disease (FTD-MND) [15].

The outlook of ALS and FTD as distinct nosological entities has progressively changed in the last decade due to genetic breakthroughs. Over this time period, more than 50 genes have been associated with ALS and FTD [16–18]. Mutations in at least 44 loci have been linked to ALS and, correspondingly, at least 14 loci have been linked to FTD. At present, the four major ALS-associated genes are the chromosome 9 open reading frame 72 (*C9ORF72*) [19, 20], Cu-Zn superoxide dismutase 1 (*SOD1*) [21], TAR DNA-binding protein 43 (*TARDBP*) [22, 23], and fusion in malignant liposarcoma/translocation in liposarcoma (*FUS/TLS*) [24, 25], in addition to at least other 40 genes [26]. On the other hand, mutations three main genes (MAPT, GRN and C9ORF72) are found in 60% of familial FTD cases, whereas rare mutations (<5%) have been found in other 11 genes [8, 27, 28].

**Fig. 1:**
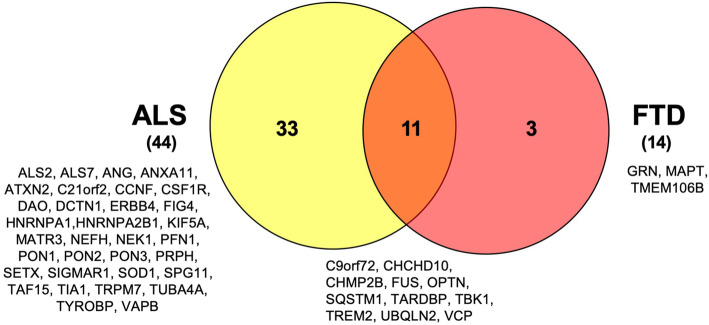
Genetic overlap between ALS and FTD. To this date, more than 50 genes have been associated with ALS and FTD. The Venn diagram summarizes the number of specifically altered genes in each disease and the observed overlap between the two pathologies

These findings have contributed to shed light on the major biological processes altered in ALS and FTD/FTLD pathologies (Table 1) [29]. On the other hand, as the genes associated with these diseases were fleshed out, it was apparent that ALS and FTD share pathophysiological processes. In fact, the intersection of the gene sets implicated in the pathogenesis of ALS and FTD shows that at least 11 susceptibility genes are in common between these two disorders (Fig. 1). This genetic observation makes the molecular overlap particularly striking and strengthens the current notion that ALS and FTD share common pathogenic mechanisms.

**Table 1:** Main genes and cellular components/processes implicated in the pathogenesis of ALS and FTD

RNA metabolism	TARDBP, FUS, hnRNPA1, hnRNPA2B1, MATR3, ATXN2, TAF15, SETX, EWSR1, ELP3, ANG
Protein trafficking and Proteostasis	C9ORF72, CHMP2B, FIG 4, TBK1, UBQLN2, SQSTM1, SIGMAR1, OPTN, VCP, ALS2, VAPB
DNA repair	EWSR1, FUS, SETX, TAF15, TARDBP
Mitochondria and Oxidative stress:	SOD1, CHCHD10, C19ORF12
Immune response/Inflammation:	GRN, TREM2, TYROBP
Stress granule assembly	ATXN2, C9ORF72, MAPT
Glia and Neurons metabolism	GRN, SIGMAR1, SOD1, TREM2, TYROBP
Vacuolar transport	C9ORF72, CHMP2B, GRN, TMEM106B, VCP, OPTN, UNC13A
Axo-dendritic transport	KIF5A, MAPT, SPG11

## No gene is an island by itself

If we analyze more thoroughly the genes implicated in the pathogenesis of ALS and FTD it is interesting to note the intricate network of coexpressions, interactions and pathways interconnecting most of the players (Supplementary Figures 1, 2, 3 and 4). In fact, a prediction of gene functions and connections carried out by using the Genemania online tool [30, 31] suggests that a large number of the susceptibility genes are linked through physical protein-protein interactions (44.57%), and are co-expressed (26.29%), whereas few gene share pathways (7.42%), or show predicted functional interactions based on orthology (6.55%).

Altogether these observations support the hypothesis that such complex diseases as ALS and FTD are likely caused not only by the simple alteration of a single gene and that these pathologies are the result of alteration of multiple genes clustered in the same pathways or in connected pathways, caused by the triggering of a “domino effect” propagating the initial pathological signal to the other physical or functional partners. In this line, TDP-43 and the related TDP-43 proteinopathies are an exemplary case. It is now well established that clinically and neuropathologically, ALS and FTD or its pathological substrate FTLD may be seen as two ends of a spectrum [13, 15, 32] as well as representing a connection with several other neuromuscular diseases [33].

Until recently, the pathological protein/s in these inclusion bodies of ALS and FTLD with ubiquitin-immunoreactive inclusions was thought to be an unknown ubiquitinated protein. Subsequently, TDP-43 was identified as the major component of the cytoplasmic inclusions of both ALS and FTLD neurons and glia [34, 35]. Deeper biochemical analysis of purified neuronal cytoplasmic inclusions has shown that pathologic TDP-43 is not only ubiquitinated, but also hyperphosphorylated, and aggregates as abnormal C-terminal fragments [23]. Phosphorylated TDP-43 intraneuronal inclusions in ALS are found within the motor cortex, brainstem motor nuclei, cranial nerve nuclei V, VII, and X-XII, and in spinal cord motor neurons. It is still unclear whether TDP-43 aggregation might represent a primary event in ALS pathogenesis or rather an epiphenomenon secondary to other pathological processes.

In addition to ALS, TDP-43 proteinopathy now also constitutes 45% of all FTLD molecular pathologies [36] (Table 2). Approximately 50% of all cases contain abnormal tau (namely, tauopathies) and the residual group of about 5% of cases is characterized by abnormal FUS (FUS proteinopathies) [61].

**Table 2:** ALS-FTD genotype/phenotype correlations for genes and presence of TDP-43 inclusions

Gene	Genetic overlap ALS/FTD	TDP inclusions	References
SETX	ALS	Yes	[37]
ATXN2	ALS	Yes	[38]
SOD1	ALS	Yes	[39, 40]
VABP	ALS	NR	--
ALS2	ALS	NR	--
ANG	ALS	Yes	[41]
SQSTM1	ALS	Yes	[42]
C21ORF2	ALS	NR	–
MATR3	ALS	Yes	[43]
EWSR1	ALS	NR	--
TAF15	ALS	NR	--
HNRPA1	ALS	Yes	[44, 45]
HNRNPA2B1	ALS	Yes	[44, 45]
OPTN	ALS	Yes	[46]
TUBA4A	ALS-FTD	NR	[47]
TARDBP	ALS-FTD	Yes	[48, 49]
C9ORF72	ALS-FTD	Yes	[50, 51]
DCTN1	ALS-FTD	Yes	[52]
TUBA4A	ALS-FTD	NR	[47]
TBK1	ALS-FTD	Yes	[53]
CHCHD10	ALS-FTD	Yes	[54]
CCNF	ALS-FTD	Yes	[55]
FUS	ALS>FTD	NR	[24, 25]
UBQLN2	ALS>FTD	Yes	[56]
SIGMAR1	ALS>FTD	NR	--
TIA1	ALS>FTD	NR	--
CHMP2B	FTD>ALS	NR	[56, 57]
VCP	FTD>ALS	Yes	[58]
GRN	FTD	Yes	[59]
MAPT	FTD	NR	--
TMEM106B	FTD	Yes	[60]

The evidence for mutations linking each gene to the ALS-FTD spectrum is reported (ALS only; FTD only; both ALS-FTD; majority of ALS cases: ALS>FTD; majority of ALS cases: FTD>ALS). *NR* not reported

Importantly, co-morbidity of TDP-43 proteinopathy seems not to be limited to ALS/FTLD but it has also been detected in several other neurological disorders, such as Alzheimer's disease (about 75% of cases with hippocampal sclerosis, 31% without hippocampal sclerosis, 30% with dementia with Lewy bodies), Parkinson's disease (about 7% and 19% of PD with dementia), dementia Parkinsonism ALS complex of Guam (G-PDC), Pick's disease, corticobasal degeneration and progressive supranuclear palsy (PSP) [62–70]. Furthermore, recent studies reported the altered expression and mislocalization of TDP-43 in brain tissues obtained from a Niemann Pick C (NPC) patient and in NPC cellular models [71].

## Disease modifier factors

**Fig. 2:**
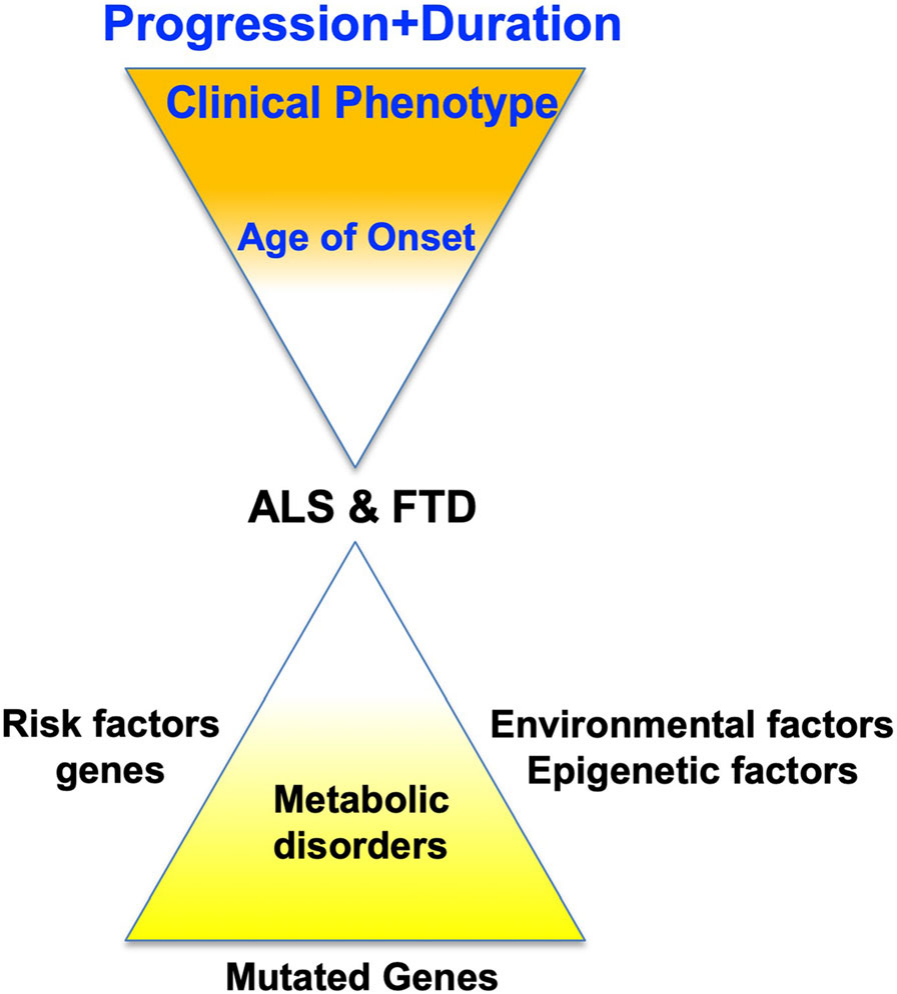
Factors affecting the disease- and clinical- phenotypes of ALS and FTD pathologies. Schematic diagram of the various exogenous and endogenous factors potentially affecting the age at onset, the disease phenotypes, as well as the clinical phenotypes

Genetic mutations are not sufficient to explain the onset and the progression rates of ALS and FTD patients. The common delayed onset of both pathologies [72], and the observation that only specific cellular types are affected suggests that the vulnerability of disease-specific neurons is not induced just by simple genetic factors. Additional environmental, metabolic and genetic factors and modifiers have to be brought into play to explain and increase the complexity of ALS and FTD etiopathogenesis (Fig. 2).

Regarding the environmental factors, elevated heavy metal levels stand out not only as risk factors for ALS but have also been proposed to correlate with ALS progression [73, 74].

It is interesting to report that different epigenetic mechanisms influencing the expression of C9ORF72, SOD1, GRN, VEGF, and GLT1 have been implicated in the pathogenesis of these disease [75, 76]. While the promoters of SOD1, VEGF, and GLT1 genes have been found to be unmethylated in ALS patients [77, 78], hypermethylation of GRN promoter has been reported in FTD subjects and the observed lower mRNA levels lend support to its correlation with GRN haploinsufficiency and FTD pathogenesis [77, 78]. On the other hand, the role of C9ORF72 gene methylation in ALS and FTD pathogenesis is still unclear. Intriguingly, only 10–30% of c9FTD/ALS subjects present hypermethylation of the C9ORF72 promoter possibly correlated with a decreased C9ORF72 expression.

Although other epigenetic modifications, such as histone and miRNA alterations have been reported in ALS and FTD patients [76], we are still lacking a clear picture of the impact of all the epigenetic findings on the clinical aspects of these pathologies.

Other intriguing lines of research evaluated some metabolic parameters as disease-modifiers that can impact the clinical course in ALS. For example, recent investigations have shown that high-risk cardiovascular profiles such as a high body mass index (BMI) or diabetes mellitus type 2 might be protective for ALS patients and may act by delaying the onset and/or by slowing down clinical progression of the disease [79–85]. Altogether, these finding suggest that caution should be exercised when comorbidities and risk factors are evaluated as prognostic factor in such as complex diseases as ALS and FTD. In particular, a better characterization of the eventual association between the clinicopathological features of the patients and a particular metabolic disorder might be helpful to understand if and how metabolic disorders can influence the subcellular localization, aggregation, and phosphorylation of some of pathogenic proteins, or if the metabolic disorders might rather modulate the toxic effects downstream of these events [81]. In addition, these observations open potentially the way to nutrition, dietary supplements (in particular, antioxidant) and lifestyle interventions as potential strategies for modifying the course of the disease [86–88]. In this line, recent studies have suggested that a high-calorie diet might be an effective treatment for ALS by inducing a hypermetabolic state that counters the increased resting energy expenditure (REE) observed in ALS patients [89–91].

## Disease modifier genes

On top of the disease-associated genes, an additional level of complexity is added by the existence of modifier genes. In fact, part of the disease and clinical phenotypes can be ascribed to the action of genes other than those directly implicated in the pathologies (Table 3).

**Table 3:** Effect of the genetic modifiers of ALS and FTD

GRN	SORT1	rs646776 C-minor allele => decreased GRN plasma expression	[92, 93]
GRN	TMEM106B	rs1990622 C-minor allele => lower risk (older age at onset)	[94, 95]
C9ORF72	TMEM106B	rs1990622 C-minor allele => earlier onset	[96]
		rs3173615 G minor allele => Homozygosity protects from developing FTD but not from developing MND	[97]
--	TMEM106B	rs1990622 T-major allele => poorer cognitive performance in FTLD-TDP patients not in ALS patients	[98]
--	TMEM106B	rs1990622 C-minor allele => protective effect on cognitive aging	[99–101]

In this context, after identification of the first mutations in the GRN gene of FTLD patients, the high clinical variability observed among the carriers of similar GRN mutations led to hypothesize the presence of environmental and/or genetic modifiers. For example, it has been found that each copy of the rs646776 minor C allele, linked to SORT1 expression, is associated with a decreased GRN plasma levels by ∼15% [92, 93].

It has been also shown the existence of a link between GRN and TMEM106B genes. In fact, variation in TMEM106B expression seems to be associated with changes in progranulin expression: in fact, it has been found that TMEM106B rs1990622 C-minor allele is associated with a delay of FTLD onset age (lower risk) for GRN mutation carriers [94, 95]. It has been also shown the existence of a link between GRN and TMEM106B gene and variations in TMEM106B expression seem to be associated with changes in progranulin expression. TMEM106B SNPs has been also reported to be risk factors across the genetic FTLD-TDP spectrum. In addition, the rs1990622 T-major allele has been associated with later age at onset and at death, whereas the homozygosity for rs3173615 G minor allele seems to protects from developing FTD but not from developing MND [96, 97]. To make the puzzle even more challenging, the rs1990622 T-major allele has been also associated with poorer cognitive performance in FTLD-TDP but not in ALS patients [98]. On the other hands, the rs1990622 C-minor allele has been shown to have a protective effect on cognitive aging in MAPT mutation carriers [99–101].

## Present drugs

The clinical and molecular heterogeneity of ALS and FTD have represented a significant challenge for the development of effective treatments.

**Fig. 3:**
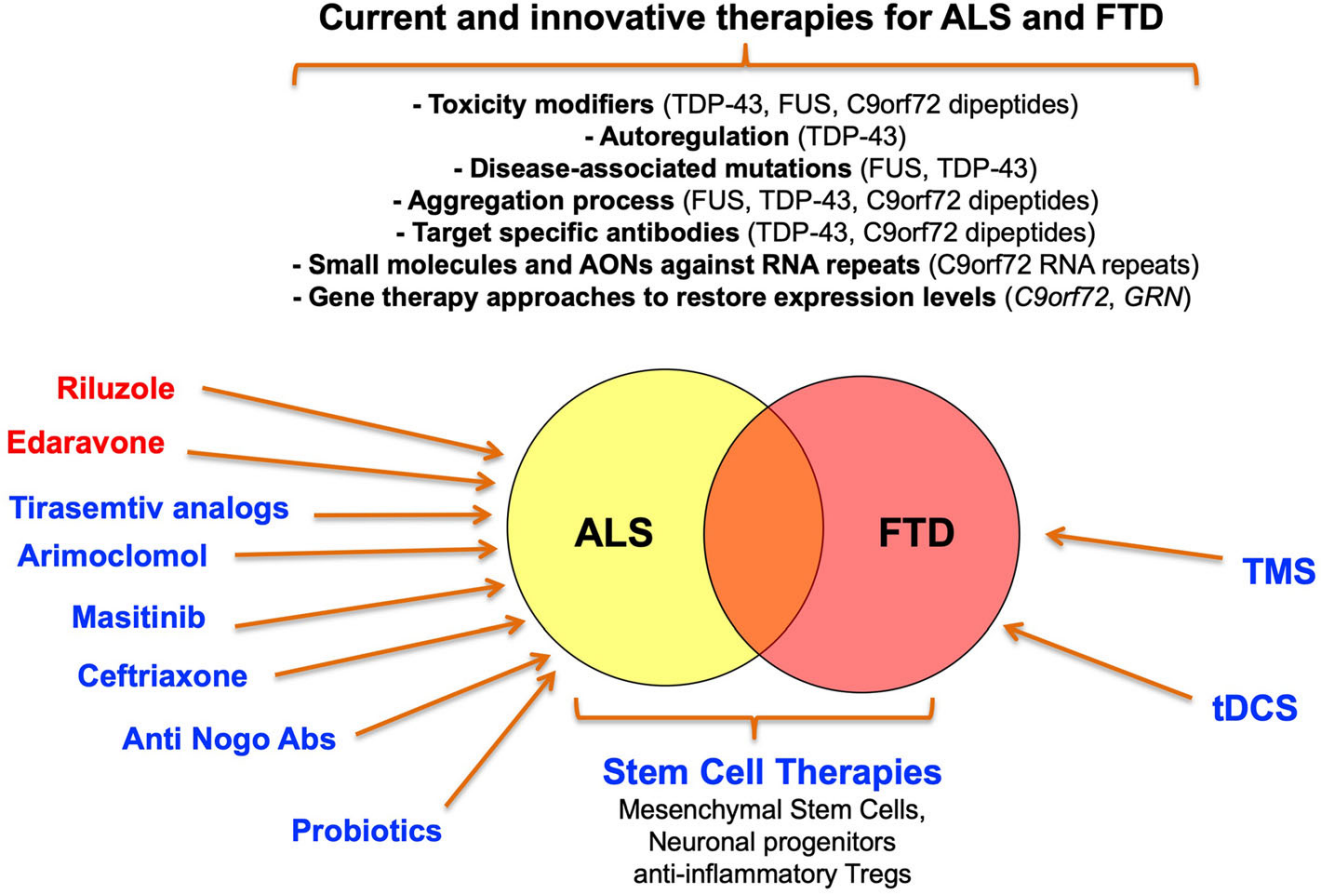
Actual and potential therapeutic approaches to treat ALS and FTD pathologies. This schematic diagram recapitulates the major pharmacological therapeutic approaches that have been tested or are currently approved for treatment of ALS patients (highlighted in red). In addition, it shows the main novel approaches that are currently under development based on recent discoveries in the field of stem-cell/gene therapy and the molecular pathology of these diseases. As shown in this figure, they range from small functional molecules and RNAs against specific protein/RNA-based targets, stem-cell approaches, to transcranial magnetic stimulation (TMS) or transcranial direct current stimulation (tDCS)

Regarding treatments for FTD, although a lot of symptomatic treatments have been reported so far, it has been found that open-label studies of anticholinesterase medicines and memantine have been negative. In some cases, they may even exacerbate behavioral symptoms [102, 103]. Concerning selective serotonin reuptake inhibitors and antipsychotic therapies these treatments can be helpful in the management of mood and behavioral features in individual patients [102]. Tauopathy has become a target for novel disease-modifying treatments such as Methylene Blue that is a drug in Phase III clinical trials. This compound was found to be effective in arresting age-related cognitive decline of tau-transgenic mice but only if administered at the earliest stages [104]. Regarding ALS, although several new potential drugs are currently being tested in Phase 1 to Phase 3 clinical trials (www.clinicaltrials.gov), there are no effective therapies able to stop the progression of ALS. Riluzole, the only disease-modifying treatment shown to extend life expectancy in patients with ALS, was the first FDA drug approved for clinical use in 1995 [105]. In initial studies, it has been associated with 38,6% reduction in mortality in an efficacy trial [106], and 35% improvement in survival with the 100 mg dose in a dose – ranging trial [107]. Despite being associated with a short survival benefit of 2–3 months equating to a 9% increase in 1-year survival it still represents the most ALS-effective therapy to this date [108]. Although the main neuroprotective activity of Riluzole relies mainly on blocking of the glutamatergic excitotoxicity several other actions have been proposed. These include inactivation of voltage-dependent sodium channels and interference with signal transduction events following interaction of neurotransmitters to excitatory amino acid receptors [109]. Only after 22 years, in 2017, the USA FDA approved the clinical use of the second ALS-specific drug, Edaravone. The Edaravone (MCI-186, also known as Radicava or Radicut) is a neuroprotective drug with free radical scavenger and antioxidant properties [110]. In a double-blind, placebo controlled, Phase 2 study using intravenous Edaravone therapy in ALS patients, Akimoto and colleagues showed a decrease in primary endpoint in the Revised ALS Functional Rating Scale (ALSFRS-R) scores from baseline to 24 weeks after randomization [111]. In the recent Phase 3 study, it has been reported that Edaravone treatment is beneficial in ALS patients even after 6 months of receiving placebo, and efficacy is maintained for up to 1 year [112]. However, both drugs have a relatively small efficacy in delaying the motor function deterioration, and their effectiveness is limited during early stages of the disease [113]. Therefore, new treatments are urgently needed to improve the clinical course of both diseases. Over the years, different approaches are emerging to identify effective therapeutic strategies against ALS and FTD (schematically depicted in Fig. 3).

## Drugs on the way

### Arimoclomol

Arimoclomol is a hydroxylamine derivative able to induce heat shock protein expression [114], whose dosing was reported to improve muscle function and survival in different mouse models of motor neuron disease [115]. This compound may have multiple mechanisms of action and several lines of evidence suggest that under non-stressed conditions it may reduce the levels of protein aggregates in motor nerves (a possible cause of ALS). This is achieved by boosting expression of the heat shock proteins Hsp70 and Hsp90 [116]. In addition, Arimoclomol was recently found to induce a HSF1-mediated reduction of the TDP-43 aggregates [117]. Finally, in order to evaluate the efficacy and safety of Arimoclomol, a Phase 3 randomized, double blind, placebo-controlled trial is currently underway (NCT03491462) after a Phase 2 trial suggested a possible therapeutic benefit of this molecule (NCT00706147) [118].

### Ceftriaxone

Ceftriaxone is a third generation of cephalosporin antibiotic, with ability to cross the blood brain barrier (BBB) and to exert neuroprotection in different animal models. This activity seems to rely on induction of the astrocytic glutamate transporter EAAT2 expression in humans, and GLT1 glutamate transporter expression in rodents. Apparently, overexpression of glutamate transporters can boost the clearance of synaptic glutamate and protects neurons from glutamate-mediated excitotoxicity, that is thought to be critical in the pathogenesis of ALS [119]. Along this line of evidence, low expression of the glutamate transporter EAAT2 has been reported in ALS animal models as well as in human post-mortem tissue [120]. Unfortunately, this compound did not show efficacy at Phase III clinical trials [121], most likely due to already well-established disease.

## Masitinib and cannabinoids

Masitinib is a selective tyrosine kinase inhibitor, with anti-inflammatory activity deriving from its ability to counteract degranulation of the mast cells. In addition, it can also inhibit microglia proliferation and activation [122]. This compound has entered Phase 3 trials for ALS in 2017, as it was observed in a Phase 2 trial that it could be of benefit to ALS patients as an add-on to Riluzole [123]. Regarding anti-inflammatory effects, it has to be noted that cannabinoids have also been proposed to be useful in the treatment of ALS due to their anti-inflammatory, anti-oxidant, and anti-excitotoxic properties [124, 125]. However, a recent meta-analyses of the studies conducted so far on animal models has concluded that more standardized studies should be performed before supporting the treatment of ALS patients with these compounds [126].

## Tirasemtiv and structural analogs

Tirasemtiv is a troponin activator with the ability to make troponin more sensitive to calcium and consequently counteract muscle weakness, that is the most common initial symptom in ALS [127]. Tirasemtiv was the subject of a Phase 3 clinical trial program in ALS patients [128, 129] (NCT02496767) but the results were disappointing, as reported in recent congress communications. However, research in this area is still currently evolving with the development of structural analogs of Tirasemtiv such as CK-2066260 [130] and, especially another skeletal muscle activator CK-2127107 [131] called Reldesemtiv has entered Phase 2 trial (NCT03160898) with, hopefully more promising results.

## Stem-cell based approaches

Stem cell therapy aimed at counteracting immune dysregulation is another promising novel treatment for several autoimmune diseases (such as rheumatoid arthritis, multiple sclerosis, lupus) [132] as well as for ALS [133]. Initial attempts using neural stem cells in a Phase 2 clinical trial (NCT01730716) did not seem to benefit patients [134]. Therefore, starting from the observation that neurotrophic growth factors seem to extend the survival of motor neurons in ALS [135, 136] a culture-medium based method was used to induce mesenchymal stem cells (MSCs) to secrete neurotrophic growth factors (NTFs) [137].

The intrathecal (i.t.) or intramuscular (i.m.) administration of autologous MSC-NTF cells has been shown to be safe and to provide possible clinical advantages for ALS patients and the potential positive outcomes are supported by other clinical studies [138–140]. However, these results should be further validated by recruiting a larger number of patients and by including a placebo control. Interestingly, it has been suggested that MSCs might modulate the inflammatory responses in ALS patients by increasing the levels of anti-inflammatory cytokines produced by activated regulatory T lymphocytes (Treg) [141, 142]. A Phase 1 study is currently in progress to determine the efficacy of these cells against disease progression [143].

## Immunological approaches

Recent studies have shown peculiar differences in the inflammatory responses underlying the pathogenesis of ALS and FTD [11, 144]. These researches pave the way to the approaches on the similarities in the immunological mechanisms of ALS and FTD with other chronic diseases where flogosis plays a critical pathogenetic role [145]. These observations explain current efforts of drug-repositioning with a series of NIH clinical trials aimed at proving the effectiveness on ALS of agents used for the treatment of Rheumatoid arthritis and Multiple sclerosis. However, although some encouraging preliminary reports were observed in animal models with regards to safety and tolerability, no clear improvements for ALS has been found [145].

At the moment, another ALS-specific immunological therapy seemed to be more promising. It was shown, in fact, that peripheral nervous system injury is associated with inhibition of the axonal growth in mammals, through activation of the reticulon 4 receptor (RTN4R, or Nogo receptor) [146]. NogoA is one of the three isoforms of the Nogo protein acting as a neurite outgrowth inhibitor [147]. It is also localized on macrophages and, following Wallerian degeneration, it has a crucial role for the clearance of these cells from the site of injury. NogoA seems to be overexpressed in the skeletal muscle of ALS patients [148]. Therefore, the efficacy of an anti-NogoA antibody (ozanezumab) to slow down ALS progression was tested but no difference in disease progression (ALSFRS-R) or in survival was observed [149].

## The therapeutic potential of the gut microbiota

One of the most prominent scientific breakthroughs of the last decade is the demonstration of the link between the gut microbiota and the brain based on the growing number of studies reporting causal effects of the gut microbiota on the brain. Taken together, these studies suggest that the microbiota might influence behavior and play a role in the pathogenesis of several neurological disorders [150–152]. In this context, preclinical studies have demonstrated that there is a difference in the microbiota profile in ALS patients. The results of these studies have suggested that such modifications can alter the gut microbiota brain axis and induce gastrointestinal dysfunction in ALS patients [153, 154]. Notwithstanding the limitation of the studies (in particular, the small panel of human samples and uncertainty whether dysbiosis is primary or secondary to dietary changes in ALS), the role of the gut microbiota in ALS stays on the cutting edge for developing therapies aimed at improving gut dysbiosis as well as the course of the disease using probiotics and prebiotics, both from a prophylactic and therapeutic points of view. Regarding this issue, it is worth to mention a recent study that demonstrates how, in an ALS-prone Sod1 transgenic mouse model, the course of the ALS-like disease can be modulated by administration of specific gut microbial strains. Apparently, administration of Akkermansia muciniphila was associated with improvements of symptoms and prolonged survival whereas Ruminococcus torques and Parabacteroides distasonis were associated with an exacerbation of disease progression. In addition, preliminary analyses of human gut microbiota further supported the hypothesis that Akkermansia might play a role in the progression of human ALS, an exciting discovery [155]. Although more studies are necessary, it is feasible to predict that the management of the gut microbiota might represent a further strategy helpful to prevent or alleviate the symptoms of this neurodegenerative disease.

## Therapeutic approaches based on non-invasive brain stimulation

An alternative and innovative approach to pharmacological therapies is provided by non-invasive brain stimulation approaches, represented by transcranial magnetic stimulation (TMS) or transcranial direct current stimulation (tDCS) to slow down clinical progression in patients with ALS. Recent studies have shown limited but promising results on muscle strength, quality of life and neurophysiological measures in ALS patients treated with these options [156, 157]. In the same view, beneficial effect of targeted language training in combination with brain stimulation was demonstrated in agrammatic variant of PPA patients [158].

## Therapeutic approaches based on genetic factors

The identification of dominant mutations in TDP-43 and FUS/TLS genes represented the starting point to highlight alterations of common pathogenic pathways in both ALS and FTD mediated by dysfunctions in RNA metabolism [159]. TDP-43 and FUS/TLS have similar structures and are implicated in several steps of mRNA and miRNA processing. On one hand, TDP-43 interacts with over 6000 RNA targets and plays a role during all phases of gene expression. It can act as a transcriptional repressor by direct DNA binding [160] and regulator pre-mRNA splicing, microRNA biogenesis, as well as RNA transport and translation [161]. Conversely, FUS/TLS binds to a single or double-stranded DNA and RNA and promotes or represses transcription and affecting splicing by interacting with RNA polymerase II [162, 163]. Both TDP-43 and FUS/TLS can modify the function of stress granules and regulate synaptic function in neurons, a feature that might represent a crucial link with the pathology [164].

Moreover, a shared characteristic of ALS/FTD is a reactive gliosis [165]. This is an unspecific proliferation of glial cells in injured brain regions characterized by microglial proliferation and astrocytic hypertrophy. Interestingly, gliosis is not the only main feature of the brain regions that show neuronal loss and inclusion pathology. In addition, glia can further promote neurodegeneration by releasing toxic compounds and by decreasing its clearance ability [166].

### TDP-43

The lack of proper models of TDP-43 aggregation (that might allow to study the mechanism of formation, and their impact on other cellular components, as well as on cell metabolism) hampers full comprehension of the role of this nuclear factor in the pathogenesis of ALS and FTD [34]. Improving characterization of the mechanisms underlying the activities of these proteins, especially TDP-43, C9ORF72, and FUS/TLS is the main logical approach to better unravel the pathogenesis of ALS and FTLD-TDP. This is currently being done together or in parallel with investigating the relevance of common pathological pathways. For example, perturbation in neurotransmitters has been demonstrated in ALS as well as in FTLD, in particular with glutamate-driven excitotoxicity [160]. This pathway is supposed to be involved in ALS pathophysiology [167] and, more recently, also in FTLD [168, 169].

One still open question in the TDP-43 field has always been to determine whether the pathology caused by this protein was through a mechanism of loss-of-function or gain-of-function (bearing in mind that both mechanisms are not necessarily mutually exclusive and may be acting at the same time or at different stages in disease) [170, 171].

At the loss of function level, aggregates are able to sequester endogenous TDP-43 depleting its nuclear levels and inducing loss of function at the RNA processing level [172]. Importantly, neurodegeneration has recently been observed in selected neuronal populations that are affected early during disease even in the absence of TDP-43 aggregation, an observation suggesting that loss of function may be present even at early stages of disease [173]. No apparent direct cellular toxicity of the aggregates seems to be present beyond the lack of functional TDP-43 although some reports using bacterially-made TDP-43 aggregates have suggested that some degree of toxicity may be present due to alteration of calcium homeostasis [174]. Regarding therapy, therefore, the engineering of protein “disaggregases”, such as Hsp104, that can free and eventually reactivate TDP-43 trapped in the inclusions, could represent a valuable therapy for ALS and FTD patients [175, 176].

As TDP-43 is a ubiquitous protein that plays a general role in cellular and developmental processes of higher organisms, it may be difficult to target this protein in a generalized manner. One possibility, therefore, might be to target regions that are important to trigger the aggregation process, such as the RNA Recognition Motif 1 (RRM-1) that was shown to interact directly with p65-NF-kB [177]. Interestingly, it has been recently shown that antibodies against TDP-43 RRM1, able to disrupt this interaction, can reduce the neuroinflammation and the motor defects in mice that express an ALS-linked TDP-43 mutation [178].

Alternatively, targeting eventual mutations in the sequence of this protein might represent a parallel alternative therapeutic option [179]. To this date, the study of various patient populations has identified more than 50 TDP-43 mutations associated predominantly with ALS [172] and occasionally also in FTLD [180]. Moreover, it is now clear that a significant number of mutations affect other disease-associated proteins such as FUS, hnRNPA1, hnRNPA2/B1, MATR3, and TIA1 [45] with the emerging picture that most of these mutations may lead to altered stress granule (SG) dynamics [181]. Indeed, a growing body of evidence suggests that inhibiting stress granule formation may be a viable therapeutic approach to suppress TDP-43 mediated toxicity [182].

Finally, it may also be possible to target selected TDP-43 RNA processing events that become disrupted by loss of function [183–185] or other functional modifiers of its toxicity such as hnRNP proteins [186], Ataxin-2 [187], matrix metalloproteinase 9 [188], TCERG1 [189], cellular kinases [190], small molecule inhibitors of translational factors [191], and even the *TARDBP* autoregulatory process at the pre-mRNA level [192].

### C9ORF72

The hexanucleotide G_4_C_2,_ repeat expansion within the *C9ORF72* gene has been recently identified as the most frequent genetic cause of both ALS and FTD [19, 20]. The pathogenic hexanucleotide length (>30 repeats) is present in about 10% of all ALS patients [193].

Focal neuronal loss, gliosis and neuronal cytoplasmic TDP-43 inclusions are the characteristic pathological findings in both C9ORF+ ALS and FTLD cases. Among the TDP-43 proteinopathies, the peculiarity of C9ORF72 pathology is that the inclusion bodies also contain dipeptide repeat polymers (DPR) and both loss- and gain- of function (LOF and GOF) of the C9ORF72 protein have been suggested to cause neurodegeneration [194, 195].

Regarding possible functions of C9ORF72, in vitro studies aimed at characterizing the effects of LOF have suggested a role in endosomal and autophagic pathways [196]. In this line, although still to be tested, C9ORF72 haploinsufficiency might eventually be addressed by gene therapy approaches [197].

On the other hand, the finding of both repeat RNA and protein aggregates in post-mortem brain supports the toxic GOF hypothesis [198]. Repeat-containing RNA aggregates, or RNA foci, have been found to trap proteins implicated in RNA splicing, editing, nuclear export, and nuclear function [199]. An alternative mechanism to RNA binding protein sequestration is associated with generation of DPRs by repeat-associated non-ATG translation (RAN), that generate neuronal inclusions with a distribution different from that of the TDP-43 pathology [200]. Small compounds targeting the peculiar G-quadruplex structure of C9ORF72 repeats have successfully used to reduced RNA foci burden and the levels of DPRs in patient iPSC-derived motor and cortical neurons [201]. Likewise to TDP-43 pathology modifiers, the screening for repressors of C9ORF72 toxicity has recently led to identify: 1) the cellular factor DDX3X as capable of inhibiting non-AUG translation of the C9ORF72 dipeptides [202]; 2) several members of the karyopherin family as capable of suppressing dipeptide toxicity [203]; 3) nuclear pore complex component Ref1 [204] and human RanGAP1 [205]. Alternatively, the development of antibodies against selected dipeptides such as GP has been shown capable of inhibiting their cell-to-cell transmission and aggregation [206].

Finally, the use of oligonucleotide-based antisense aimed at decreasing aberrant RNAs expression represents one of the more successful approaches for the treatment of various neurodegenerative disorders [207, 208]. Therefore, it has been suggested that such a strategy might be useful to reduce the levels of mutant C9ORF72 transcripts [209] and successful targeting of mutant C9ORF72 transcripts in different ALS model systems has already been described [210]. Moreover, artificial miRNAs targeting mutant C9ORF72 have been shown to be able to reduce the GOF caused by the repeat-containing transcripts [211]. The therapeutic potential of these approaches, however, still remains to be tested.

### FUS

Similarly to TDP-43, FUS is a predominantly nuclear protein and pathological FUS inclusions are mostly localized in the cytosol [212]. Mutations in the *FUS/TLS* gene account for approximately 4% of fALS (~0.4% of all ALS) [24, 25] and FTLD [213]. Although the phenotype associated with a *FUS* mutation is variable, most patients predominantly demonstrate loss of lower motor neurons and low survival rate [214]. The mechanisms by which FUS mutations cause ALS and FTD remain controversial and have been linked to a variety of neuronal features, such as dendrite development [215], and alterations in cellular processes, such as paraspeckle formation [216]. Recently, a model has been proposed in which low-complexity domains of FUS drive its reversible assembly into membrane-free, liquid droplet and hydrogen-like structure. Since the inhibition of these fibrillar hydrogel assemblies mitigates neurotoxicity, it has been proposed as a potential therapeutic strategy in early phase ALS and ALS/FTD associated with mutations [217], and approach that could potentially be used also for other RNA binding proteins.

As with TDP-43 and other ALS genes, autophagy enhancement using small molecules has been shown to successfully reduce cytoplasmic FUS levels, to restore RBP homeostasis, and to rescue motor function in vivo [218]. In keeping with this view, it has been shown very recently that a potent HDAC inhibitor, called ACY-738, can cross the blood-brain barrier and improve the motor phenotype and life span of Transgenic *FUS*+/+ mice [219]. Finally, an early Phase 1 Clinical Trial has been started using the FDA-approved steroid medication Betamethasone (The TRANSLATE Study, NCT03707795) and the recruitment process has been just completed.

### GRN

Autosomal dominant mutations in the *GRN* gene have been implicated in up to 25% of familial FTD cases and these mutations seem to cause the disease due to haploinsufficiency [220, 221]. Mutations in GRN also result in TDP-43 neuropathology in humans, but knockout mice show little pathologically phosphorylated TDP-43 [222], thus indicating that the link between gene defect and pathology is complex. Although the specific functions of *GRN* have not been fully characterized [223], it is becoming clear that in the neuronal context the expression and function of *GRN* is an important determinant of proper neurite outgrowth and branching [224]. An obvious therapeutic strategy for carriers of GRN mutations would thus be represented by gene therapy aimed at restoring proper GRN expression levels. This strategy has not yet been tried in humans but recent promising results have shown that using Adeno Associated Vectors (AAV) capable of expressing progranulin in the brain of Grn -/- mice have the ability to improve lysosomal dysfunctions and microglial pathology [225]. Finally, with regards to disease, it is also important to note that TDP-43 loss of function caused by aberrant aggregation can also induce a mis-splicing event in Sortilin1, the neuronal receptor of Progranulin [226, 227]. In this respect, therefore, these recently identified PGRN binding receptors may aid in the development of therapeutics designed to regulate PGRN levels. More recently, it has been announced by Alector company that FDA has granted Fast Track designation to AL101 for the treatment of FTD patients with progranulin gene mutations. AL101 is an anti-sortilin human monoclonal antibody designed with the aim to rescue progranulin haploinsufficiency in the central nervous [228].

## Conclusions and perspectives

The clinical and neuropathological heterogeneity of ALS and FTD represents only the tip of the iceberg of these multifaceted diseases and many key issues remain to be fully explained, such as the reason for the selective vulnerability of cell types like specific motor neurons compared to frontal and temporal neurons as well as the influence of exogenous and endogenous modifier factors on the onset and progression of disease. Moreover, there are no appropriate biomarkers capable of accurate diagnosing and predicting disease progression. Nonetheless, it is becoming increasingly clear that the main reason of the complexity of these pathologies arises from the heterogeneity in their etiology. Approximately 90% of patients present sporadic adult-onset forms of unknown etiology. The remaining percentage of patients with genetic forms of the pathologies is characterized by a high degree of genetic heterogeneity both at allele and at locus level [17, 229]. Furthermore, several risk factors and genetic modifiers able to increase susceptibility to the diseases, or to influence the rate of progression have now been identified [17, 230]. In conclusion, the two diseases represent a pathological "continuum" possibly associated with a complex inheritance and influenced by an important interplay between genetic risks and likely environmental factors.

In familial ALS (fALS) many causative gene defects have been described (for an up-to-date list see: http://alsod.iop.kcl.ac.uk/) and in September 2019, FDA released a new guidelines on the elaboration of novel treatments for ALS, providing suggestions for the design of clinical trials and to measure the effectiveness of the potential treatments [231].

At present, the road towards an effective treatment for ALS and FTD lies still in the future. Table 4 summarizes the upstream hurdles to lead the development of novel treatments for ALS and FTD. Recent advances in our better understanding of disease and the genes that are involved in the pathology have considerably improved the outlook of developing innovative therapeutic strategies in the near future. As we have highlighted in this review, and shown in Fig. 3, these therapeutic strategies involve a number of approaches ranging from classic gene therapy to small compounds aimed at modifying aberrant splicing profiles or at reducing selected mRNA expression, as well as to administration of probiotics. Most importantly, the application of other innovative treatments based on antisense oligonucleotides (ASOs) to spinal muscular atrophy and other neurodegenerative diseases provides a solid basis for their utilization also for ALS [232]. Of course, a series of desirable goals need to be accomplished in order to improve the translation of individual genetic information into novel and eventually personalized treatments for ALS and FTD (Table 5). In particular, the development of robust animal models and protocols to minimize eventual off target effects, the optimization of ASOs' delivery across the blood-brain barrier, and the overcoming of the potential concerns with regards to the immunogenicity of the viral vectors as well as to the eventual pre-existing immunity to AAV [233, 234].

**Table 4:** Hurdles on the road of developing novel treatments for ALS and FTD

• Unknown etiology of sporadic cases.	
• High degree of heterogeneity at clinical, neuropathological level.	
• High degree of genetic heterogeneity both at allele and at locus level.	
• Unknown reason(s) for the selective vulnerability of cell types (i.e., specific motor neurons, frontal, and temporal neurons).	
• Unknown influence of exogenous factors on the onset and progression of the diseases.	
• Unknown influence of endogenous modifier factors on the onset and progression of disease.

**Table 5:** Future directions to improve the translation of individual genetic information into novel and personalized treatments for ALS and FTD

• Gaining novel insights into molecular mechanisms of ALS and FTD pathophysiology by better integration of Clinical, Neuropathological, Neuroimaging, Next-Generation Sequencing, Proteomics, Pharmacogenetics studies.	
• Characterization of common and divergent mechanisms leading to ALS and FTD.	
• Revamp of ALS and FTD disease classification system according to the novel genetic and molecular information to identify subgroups of patients that might respond to treatments at a higher (or lower) rate than the population average.	
• Identification of reliable biomarkers for diagnosing, monitoring the response to therapy, and predicting disease progression.	
• Development of robust animal models and protocols to minimize eventual off target effects.	
• Optimization of ASOs' delivery across the blood-brain barrier.	
• Decrease/bypass the viral vectors' immunogenicity and the eventual pre-existing immunity to AAV.	

For sure, the more we know about the clinical, neuropathological, genetic and molecular characteristics of patients, the better we will be able to improve ALS and FTD disease classification systems in order to identify subgroups of patients. This will hopefully put researchers on the right path to integrate the novel achievements in everyday clinical practice and the development of personalized approaches to treat specific subsets of patients, based on their particular clinico-genetic “signature”.

## Supplementary information

**Additional file 1: Supplementary Figure S1.** Physical interactions of the genes implicated in the pathogenesis of ALS and FTD. Interactions by binding among the genes shown in Fig. 1 generated by using the online web-portal Genemania (https://http://genemania.org/), gene multiple association network integration algorithm. This analysis shows the complex network of interactions existing among all the genes implicated in the pathologic processes underlying the origin and development of ALS and FTD.

**Additional file 2: Supplementary Figure S2.** Co-expression of the genes implicated in the pathogenesis of ALS and FTD. Co-expression networks of the genes shown in Fig. 1 generated by using the online web-portal Genemania (https://http://genemania.org/).

**Additional file 3: Supplementary Figure S3.** Common pathways of the genes implicated in the pathogenesis of ALS and FTD. Shared pathways among the genes shown in Fig. 1 generated by using the online web-portal Genemania (https://http://genemania.org/).

**Additional file 4: Supplementary Figure S4.** Co-localization of the genes implicated in the pathogenesis of ALS and FTD. Co-localization of the genes shown in Fig. 1 generated by using the online web-portal Genemania (https://http://genemania.org/).
