# Supplementary figures and images for: From basic research to the clinic: innovative therapies for ALS and FTD in the pipeline
Source: Mol Neurodegener. 2020 Jun 1;15:31. doi: 10.1186/s13024-020-00373-9 (PMC7268618; doi:10.1186/s13024-020-00373-9)

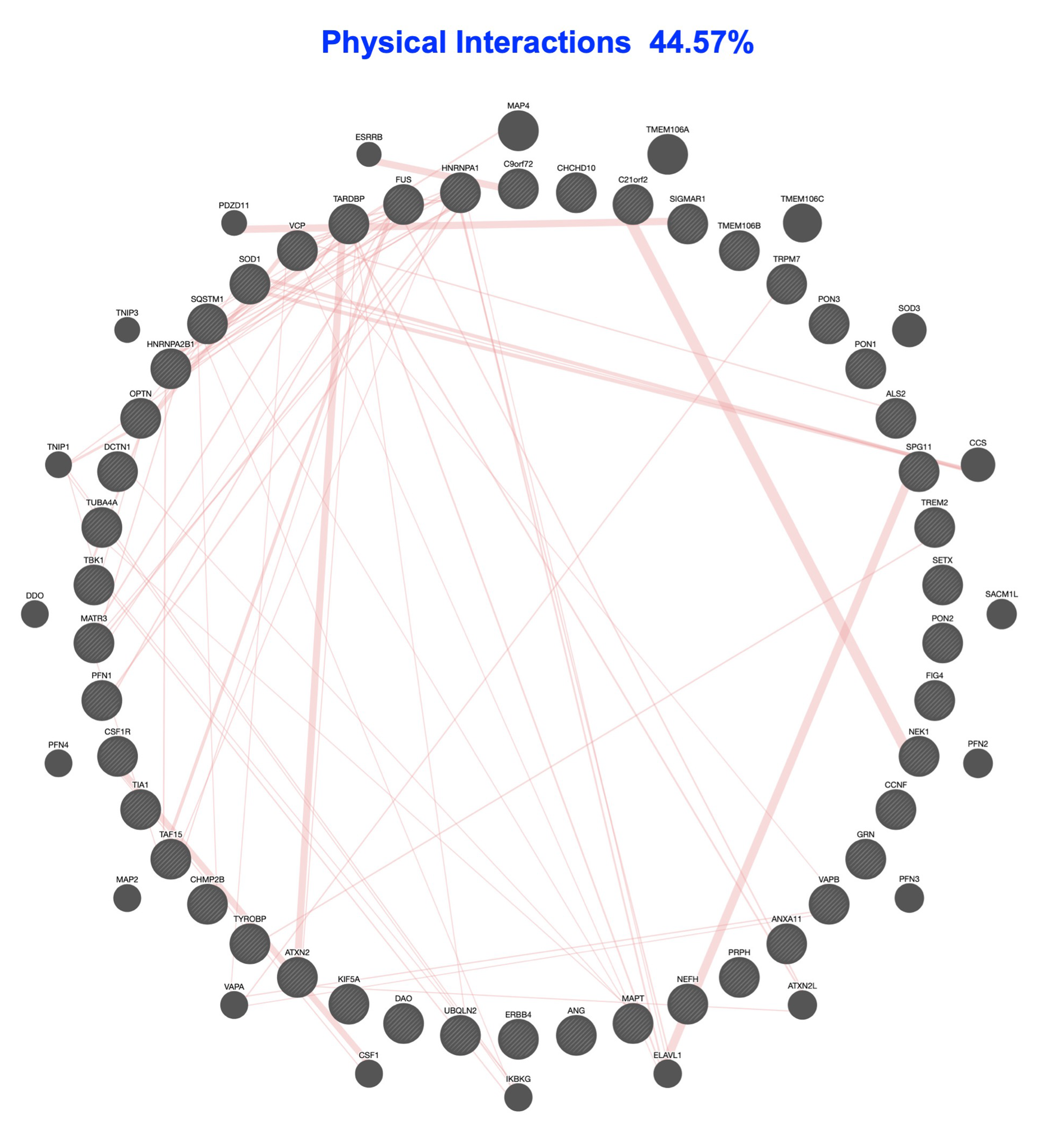

Supplement: Supplementary file 1 — Additional file 1: Supplementary Figure S1. Physical interactions of the genes implicated in the pathogenesis of ALS and FTD. Interactions by binding among the genes shown in Fig. 1 generated by using the online web-portal Genemania (https://http://genemania.org/), gene multiple association network integration algorithm. This analysis shows the complex network of interactions existing among all the genes implicated in the pathologic processes underlying the origin and development of ALS and FTD. [file 13024_2020_373_MOESM1_ESM.tif]

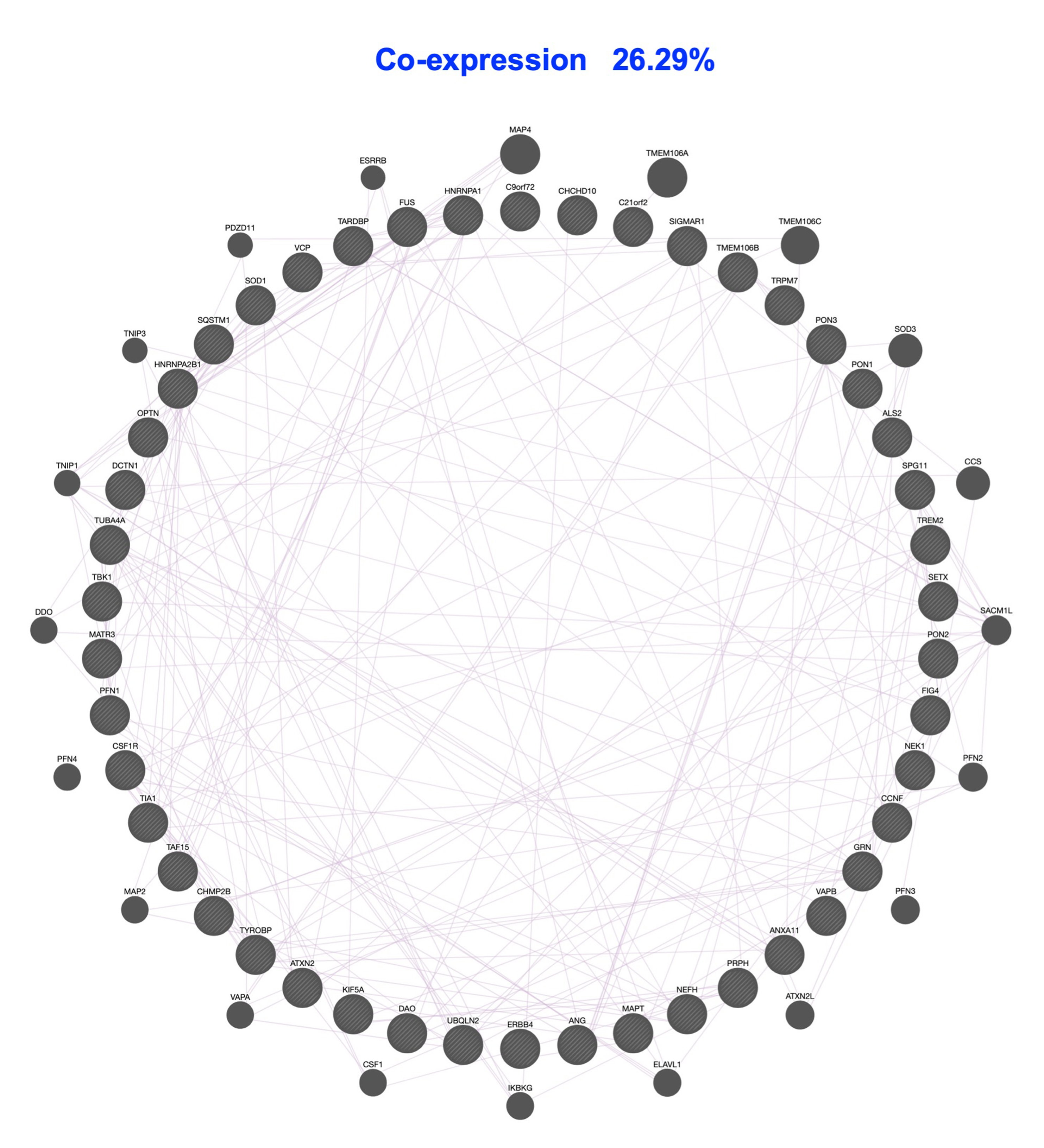

Supplement: Supplementary file 2 — Additional file 2: Supplementary Figure S2. Co-expression of the genes implicated in the pathogenesis of ALS and FTD. Co-expression networks of the genes shown in Fig. 1 generated by using the online web-portal Genemania (https://http://genemania.org/). [file 13024_2020_373_MOESM2_ESM.tif]

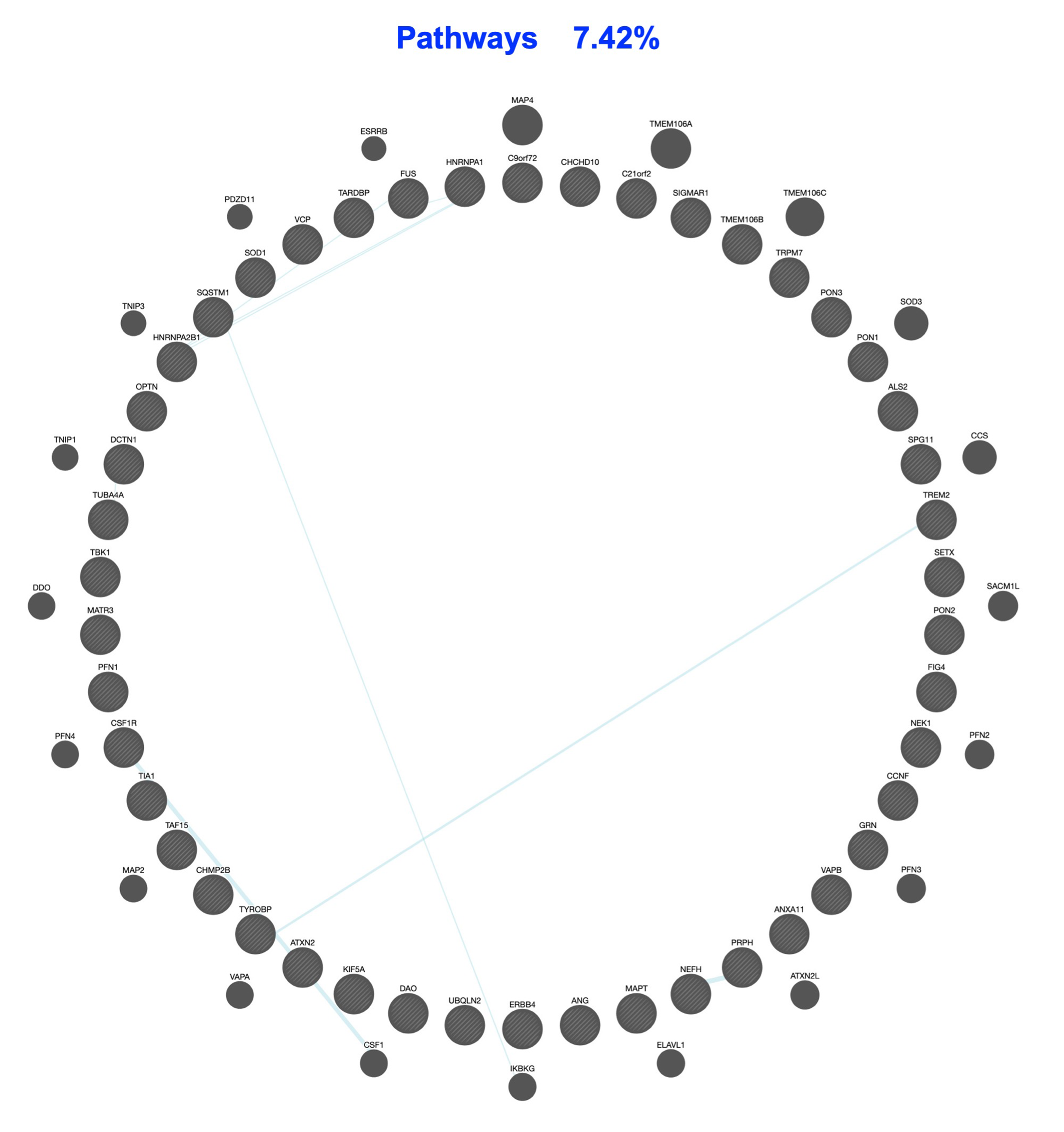

Supplement: Supplementary file 3 — Additional file 3: Supplementary Figure S3. Common pathways of the genes implicated in the pathogenesis of ALS and FTD. Shared pathways among the genes shown in Fig. 1 generated by using the online web-portal Genemania (https://http://genemania.org/). [file 13024_2020_373_MOESM3_ESM.tif]

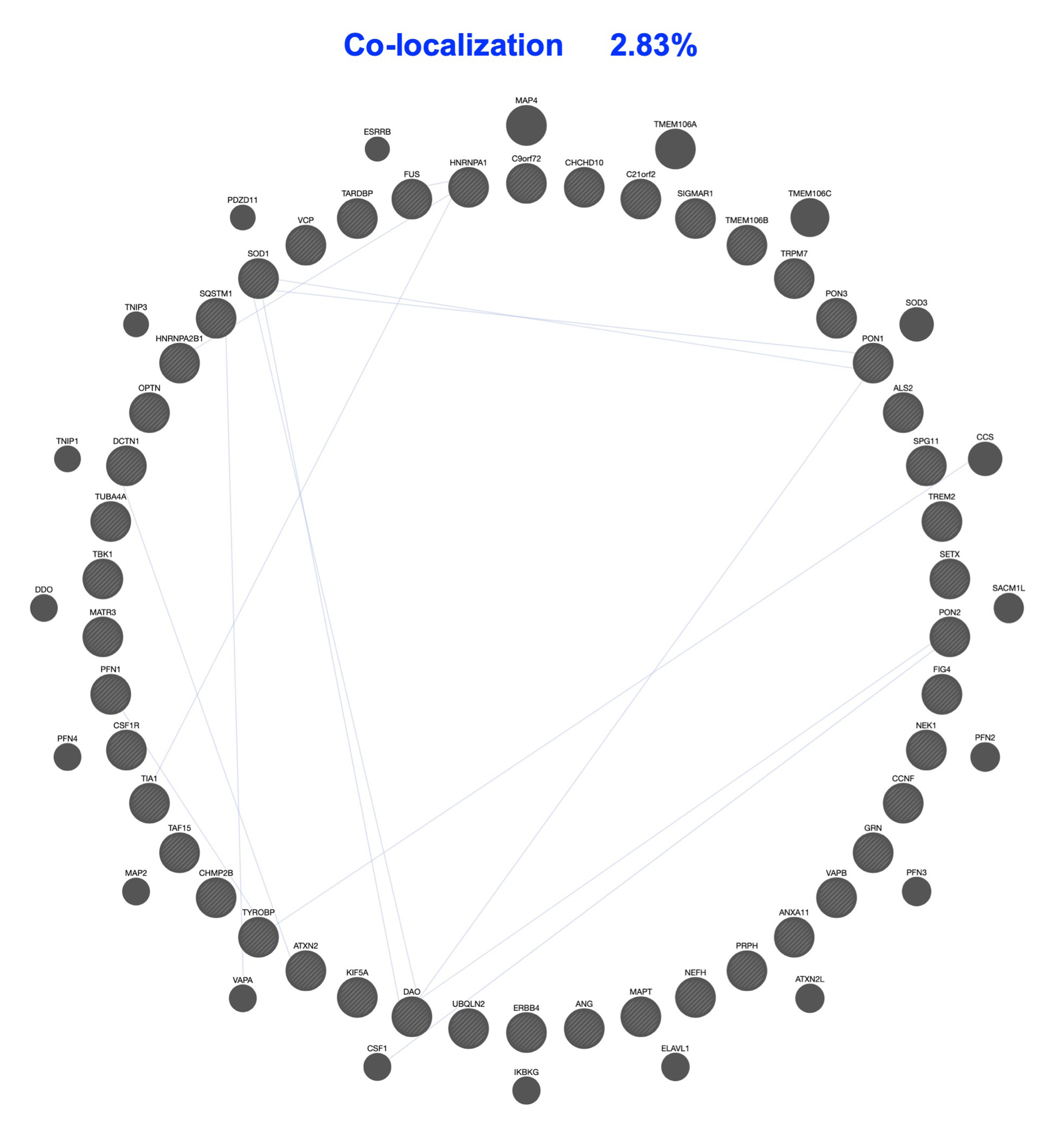

Supplement: Supplementary file 4 — Additional file 4: Supplementary Figure S4. Co-localization of the genes implicated in the pathogenesis of ALS and FTD. Co-localization of the genes shown in Fig. 1 generated by using the online web-portal Genemania (https://http://genemania.org/). [file 13024_2020_373_MOESM4_ESM.tif]
